# Obscure gastrointestinal bleeding resulting from small bowel neoplasia; A case series

**DOI:** 10.1016/j.ijscr.2019.05.006

**Published:** 2019-05-10

**Authors:** Jia Wei Teh, Amy L. Fowler, Noel E. Donlon, Waqar Khan, Iqbal Z. Khan, Michael Waldron, Kevin Barry

**Affiliations:** Department of General Surgery, Mayo University Hospital, Westport Road, Curragh, Castlebar, Co. Mayo, Ireland

**Keywords:** Obscure gastrointestinal bleeding, Small bowel, Case report, Neuroendocrine tumour, Gastrointestinal stromal tumour

## Abstract

**Introduction:**

Undiagnosed gastrointestinal bleeding may originate in the small bowel. This presents a diagnostic challenge despite the advancement in contemporary imaging. We report two cases which highlight the limitations of routine investigation for obscure gastrointestinal bleeding.

**Presentation of case:**

Patient A presented with a history of rectal bleeding, treated with interventional embolisation of caecal angiodysplasia. A diagnosis of neuroendocrine tumour (NET) was reached two years after presentation following intraoperative right hemicolectomy resection of a presumed recurrent angiodysplastic bleed. Patient B presented with recurrent melaena labelled as non-steroidal anti-inflammatory drug (NSAID) induced gastritis. After multiple endoscopic and radiological investigations, a 4.5 cm mass was visualised on imaging after three years, which was histologically proven as gastrointestinal stromal tumour (GIST) of the small bowel. Both patients experienced a delayed diagnosis despite multiple investigations and careful follow-up.

**Discussion:**

Our case series discusses the benefits and limitations of investigation for gastrointestinal bleeding and suggests a need for continued multidisciplinary input in situations where the patient presumed diagnosis remains in question.

**Conclusion:**

OGIB remains a diagnostic challenge and is attributable to small bowel pathology in 75% of cases. This suggests a need for continued investigation in situations where the patient presents multiple times despite adequate treatment for the presumed underlying condition.

## Highlights

• Obscure GI bleeding can be a challenging diagnosis and is well acknowledged.
• Perseverance is required to achieve the correct diagnosis in obscured GI bleeding.
• Diagnostic overshadowing can halt investigations and delay diagnosis.
• Multidisciplinary team input is important in the diagnosis of obscured GI bleeding.

## 1 Introduction

Upper and lower endoscopy remains the gold standard for visualising the gastrointestinal tract and allows histological confirmation of pathology [1] however imaging is limited to the second part of the duodenum in the upper GI tract and the terminal ileum in the lower GI tract. Undiagnosed gastrointestinal bleeding may be found to originate in the small bowel [2]. Further diagnostic methods, including video capsule endoscopy (VCE), double balloon enteroscopy (DBE), computed enteroscopy angiography (CTA) and magnetic resonance imaging (MRI) may be utilised to investigate causes of small bowel haemorrhage [2]. The aim of our case series is to highlight the diagnostic challenges associated with GI haemorrhage, the limitations of small bowel imaging techniques and less common pathologies which may be considered in undiagnosed GI bleeding. This case series has been reported in line with the PROCESS criteria [3]. This case series is registered in accordance with the declaration of Helsinki and exempted from ethical approval.

## 2 Presentation of case – patient A

Patient A, a 63 year old male, presented on multiple occasions with symptomatic iron deficiency anaemia and large volume rectal bleeding. His past medical history was significant for coronary artery bypass graft, atrial fibrillation, non-insulin dependent diabetes mellitus, chronic obstructive pulmonary disease and hypercholesterolaemia. He was not taking anticoagulants. He presented on four separate occasions from May to December 2015 and was investigated with multiple gastroscopy and colonoscopy, which were negative. Computed tomography of abdomen and pelvis (CT AP) highlighted widespread lymphadenopathy involving the mesenteric, retrocrural, retroperitoneal, iliac chain and inguinal regions. MRI of the small bowel was unremarkable. VCE was also non-contributory. In May 2017, he underwent a DBE which demonstrated caecal angiodysplasia, treated by interventional embolization of the ileocaecal branch.

In June 2017, he presented to the Emergency Department with ongoing rectal bleeding. On examination, he was haemodynamically stable, but pale and tachypnoeic with no signs of an acute abdomen. Urgent haematological investigations were significant for haemoglobin of 5.4 g/dL with an associated acute kidney injury (creatinine 120 μmol/L, urea 9.5 mg/dL). After transfusion of four units of red cell concentrate **(**RCC**)** and medical optimisation, he was transferred for definitive haemorrhage control. At laparotomy, an annular stricturing mass 60 cm proximal to the ileocaecal junction with associated lymphadenopathy at the root of the mesentery was demonstrated. Oncologic right hemicolectomy with side-to-side primary anastomosis was performed. Histology demonstrated a Grade 1 well differentiated neuroendocrine tumour (NET) of the ileum (T4N1M0) with mitoses of <2/10 HPF, proliferative index by Ki67 of <2% and clear resection margins.

## 3 Presentation of case – patient B

Patient B, a 66 year old gentleman, presented to Gastroenterology outpatient services in September 2013 with long standing iron deficiency anaemia (IDA) and multiple episodes of melaena. This was initially investigated with gastroscopy, colonoscopy and CT AP which were negative for pathology.

**Fig. 1:**
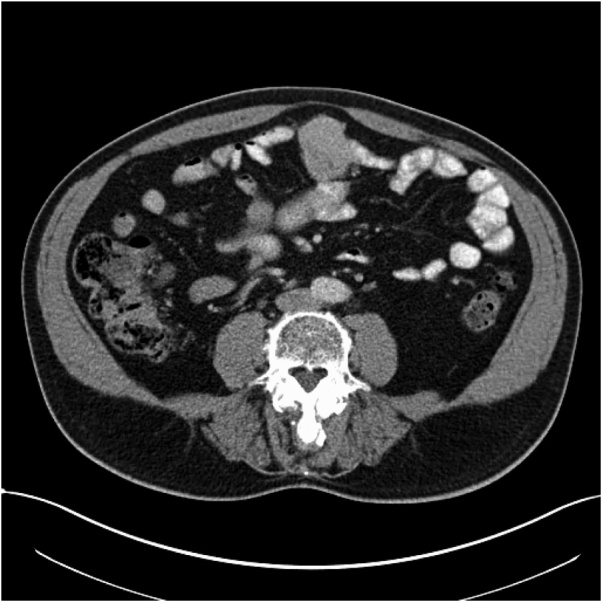
Coronal view of CT AP showing 4.5 cm lobulated mass in the anterior abdominal wall.

**Fig. 2:**
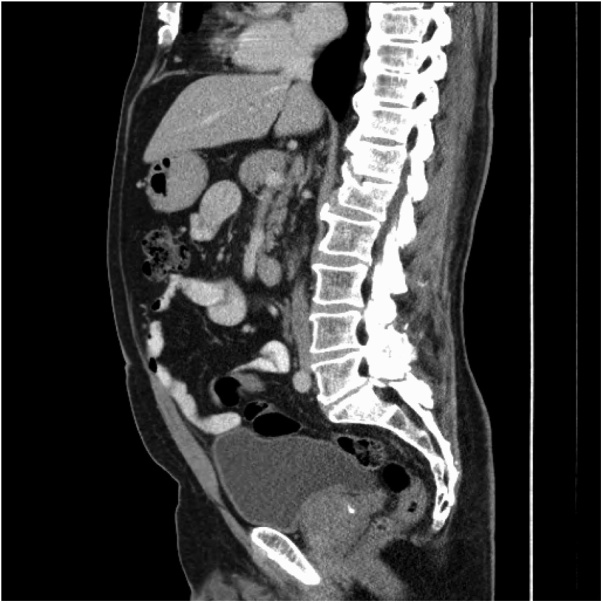
Sagittal view of CT AP showing 4.5 cm lobulated mass in the anterior abdominal wall.

In June 2016, the patient returned to the Gastroenterology outpatient clinic complaining of right sided abdominal pain, anorexia, malaise, altered bowel habit, unintentional weight loss and night sweats. Repeat gastroscopy and colonoscopy revealed antral gastritis deemed secondary to non-steroidal anti-inflammatory (NSAID) use for arthralgia. Iron studies confirmed iron deficiency anaemia (iron 6 μmol/L, transferrin 4.0 g/L, total iron binding capacity 100 μmol/L, transferrin saturation of 6%). NSAIDs were discontinued and an oral proton pump inhibitor (PPI) commenced. Repeat imaging (CT AP) for ongoing IDA in January 2017 (see Fig. 1, Fig. 2) revealed a 4.5 cm lobulated mass in the anterior abdominal wall, intimately associated with small bowel loops. He was referred for surgical input.

Ultrasound guided biopsy revealed spindle cell tumour with fascicular growth pattern, strongly positive for vimentin and C-kit and 20% positive for Ki67, confirming a diagnosis of gastrointestinal stromal tumour. In April 2018, he underwent laparoscopy, small bowel resection and primary anastomosis. Postoperative histological analysis demonstrated a 5 cm C-Kit (CD117) positive, spindle cell type gastrointestinal stromal tumour (GIST).

## 4 Discussion

Obscure GI bleeding (OGIB) is defined as overt or occult bleeding that persists after initial negative endoscopic examination [4]. Five percent of GI bleeding falls into this category [5] and in 75% of patients, pathology is within the small bowel [5]. About 5–7% of OGIB can be attributable to small bowel tumours [6,7] (Table 1).

**Table 1:** Aetiology of small bowel bleeding [8].

Aetiology of small bowel bleeding	Frequency
Mucosal vascular abnormalities	80%
Neoplasms	6–15%
Others eg. Crohn’s disease, Meckel’s diverticulum, vasculitis	10–20%

**Fig. 3:**
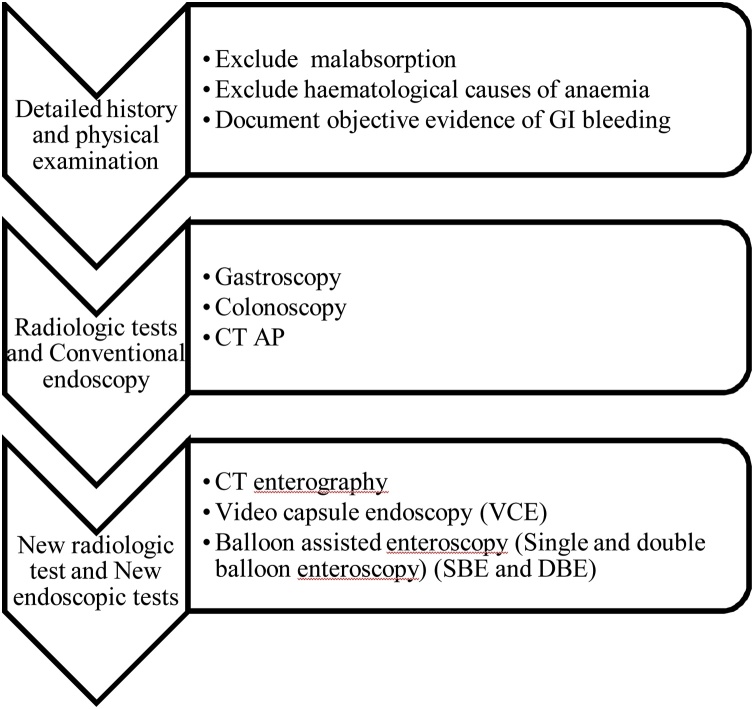
Sequence of investigation of OGIB in accordance to the ACG clinical guideline [2].

British Society of Gastroenterology (BSG) [9] and American College of Gastroenterology (ACG) [2] guidelines (Fig. 3) recommend video capsule endoscopy (VCE) for OGIB following negative gastroscopy and colonoscopy. Where VCE fails to identify the source, a second look VCE may be considered, with an additional yield of 35–75%, before investigation with double balloon enteroscopy (DBE) [9]. Intra-operative endoscopy may be indicated if diagnosis is still uncertain [9]. Multiphasic CT may also be used to guide further management [2].

A meta-analysis by Pasha et al [10] demonstrated identical diagnostic yields for VCE and DBE for small bowel vascular pathology and mass lesions, (24% and 11% respectively) and similar yields for inflammatory pathology (VCE = 15%, DBE = 16%) [10]. However, a number of studies report different outcomes for these diagnostic methods depending on the magnitude of bleeding. Pennazio et al. reported diagnostic yield of VCE in overt bleeding and occult bleeding was 92% and 44% respectively [11]. Shinozaki et al reported diagnostic yield of DBE in overt bleeding as 77% and in occult bleeding, 67% [12]. This suggests VCE may be a suitable investigation for overt bleeding and DBE more suited for occult haemorrhage.

Despite extensive evaluation, Patient A and Patient B received delayed diagnoses of small bowel neoplasm. Both VCE and DBE have limitations in their diagnostic value. VCE is operator-dependent and does not confirm histological diagnosis, with a rate of capsule retention of 1.2% [13]. DBE serves both diagnostic and therapeutic purpose. It is worth noting that it is invasive and time consuming, carrying an increased risk of intestinal perforation and bleeding [13].

Our case highlighted variability in the diagnosis of NET presenting with OGIB. Hernandez et al. reported small bowel NET causing OGIB, later diagnosed using DBE [14]. Dutta et al reported another case of small bowel NET, diagnosed during emergency laparotomy despite normal colonoscopy 6 months prior [15]. Considering that NETs are the most common malignancy in the small bowel [16], measurement of 24 -h urinary 5-HIAA and plasma chromogranin A levels may be appropriate when initial evaluations are negative [17].

Romero-Espinosa et al. described 5 cases of small intestinal GIST presenting with OGIB, concluding that CT imaging and angiography remains the main tool for diagnosis [18]. Patient B had repeated CT AP with no mass identified. Gu et al. concluded that endoscopic ultrasound (EUS) guided fine needle aspiration (FNA) could diagnose GIST accurately and efficiently [19].

## 5 Conclusion

OGIB remains a diagnostic challenge with small bowel pathology present in 75% of cases. Both patients experienced delayed diagnosis despite multiple investigations and careful follow-up. This suggests the need for continuous investigation where patients present recurrently despite adequate treatment for the initial diagnosis. NETs and GISTs are distinct in pathology and represent uncommon but important causes of gastrointestinal bleeding. Clinicians should consider unusual causes of gastrointestinal bleeding when the clinical picture is unconvincing.

## Conflicts of interest

None.

## Sources of funding

None.

## Ethical approval

Study is exempt from ethical approval.

## Consent

Written informed consent was obtained from the patients for publication of this case report and accompanying images. A copy of the written consent is available for review by the Editor-in-Chief of this journal on request.

## Author contribution

Jia Wei Teh, Amy L. Fowler, Noel E. Donlon, Waqar Khan, Iqbal Z. Khan, Michael Waldron, and Kevin Barry contributed to data acquisition, analysis, interpretation and drafting the case series.

## Registration of research studies

Research Registry - researchregistry4719.

## Guarantor

Jia Wei Teh.

## Provenance and peer review

Not commissioned, externally peer-reviewed.
